# The Human Touch: Using a Webcam to Autonomously Monitor Compliance During Visual Field Assessments

**DOI:** 10.1167/tvst.9.8.31

**Published:** 2020-07-20

**Authors:** Pete R. Jones, Giorgia Demaria, Iris Tigchelaar, Daniel S. Asfaw, David F. Edgar, Peter Campbell, Tamsin Callaghan, David P. Crabb

**Affiliations:** 1 Division of Optometry and Visual Sciences, School of Health Sciences, City, University of London, London, UK; 2 Department of Ophthalmology, University of Groningen, University Medical Center Groningen, Groningen, The Netherlands; 3 Graduate School of Medical Sciences (Research School of Behavioral and Cognitive Neurosciences), University of Groningen, Groningen, The Netherlands; 4 Ocusweep, Turku, Finland; 5 Doctoral Program in Clinical Research, University of Turku and Turku University Hospital, Turku, Finland; 6 Department of Ophthalmology, Guy's and St Thomas’ NHS Foundation Trust, London, UK

**Keywords:** affective computing, visual fields, perimetry, glaucoma, compliance, adherence, vigilance, measurement error, reliability, psychophysics, eye gaze, head pose, facial expression, OpenFace, action units, machine learning, deep learning, computer vision

## Abstract

**Purpose:**

To explore the feasibility of using various easy-to-obtain biomarkers to monitor non-compliance (measurement error) during visual field assessments.

**Methods:**

Forty-two healthy adults (42 eyes) and seven glaucoma patients (14 eyes) underwent two same-day visual field assessments. An ordinary webcam was used to compute seven potential biomarkers of task compliance, based primarily on eye gaze, head pose, and facial expression. We quantified the association between each biomarker and measurement error, as defined by (1) test–retest differences in overall test scores (mean sensitivity), and (2) failures to respond to visible stimuli on individual trials (stimuli −3 dB or more brighter than threshold).

**Results:**

In healthy eyes, three of the seven biomarkers were significantly associated with overall (test–retest) measurement error (*P* = 0.003–0.007), and at least two others exhibited possible trends (*P* = 0.052–0.060). The weighted linear sum of all seven biomarkers was associated with overall measurement error, in both healthy eyes (*r* = 0.51, *P* < 0.001) and patients (*r* = 0.65, *P* < 0.001). Five biomarkers were each associated with failures to respond to visible stimuli on individual trials (all *P* < 0.001).

**Conclusions:**

Inexpensive, autonomous measures of task compliance are associated with measurement error in visual field assessments, in terms of both the overall reliability of a test and failures to respond on particular trials (“lapses”). This could be helpful for identifying low-quality assessments and for improving assessment techniques (e.g., by discounting suspect responses or by automatically triggering comfort breaks or encouragement).

**Translational Relevance:**

This study explores a potential way of improving the reliability of visual field assessments, a crucial but notoriously unreliable clinical measure.

## Introduction

Visual field assessments are central to the diagnosis and management of many medical conditions, including glaucoma and stroke. When done well, they can yield important clinical information^1^ and have been used successfully as end points in major clinical trials.^2^ However, visual field assessments are often demanding for patients.^3^ They require sustained concentration, and patients can become bored, confused, or fatigued, sometimes leading to unreliable data.^4^^–^^6^

Previous research has focused primarily on ways to identify and discard poor-quality test data post hoc.^7^^,^^8^ What would be better, however, is if perimeters were capable of recognizing when a patient's concentration is beginning to wane. The machine could then automatically take preemptive steps to minimize the acquisition of bad data, such as by repeating trials, discounting suspect responses, pausing the test, or offering encouragement,^9^^,^^10^ just as a human clinician would do. With new machine learning and computer vision techniques, this may now be possible. For example, by using an ordinary webcam it is possible to autonomously extract real-time measures of head movements, eye gaze, and facial expressions, all of which may be indicative of test compliance (affective computing). Such signals are often used by pediatric clinicians to determine when a young patient is alert and engaged,^11^ and they could in principle be exploited likewise by automated perimeters when examining adults. This, in turn, could lead to more reliable perimetric tests, as well as fully autonomous (non-technician led) assessments of the sort necessary for home monitoring,^12^ mass screening, or rapid triage.

In the present work, we explored the feasibility of using autonomous derived biomarkers to monitor compliance. We extracted seven easy-to-obtain biomarkers and quantified the association with measurement error, as defined by (1) test–retest differences in overall test scores (mean sensitivity), and (2) failures to respond to visible stimuli on individual trials (lapses in concentration).

## Methods

### Participants and Procedure

We examined 42 eyes from 42 adults with corrected-to-normal vision (median [interquartile range, IQR] age: 26 [22–29] years), and 14 eyes from seven adults with an established diagnosis of glaucoma (69 [64–74] years of age).

**Figure 1.**
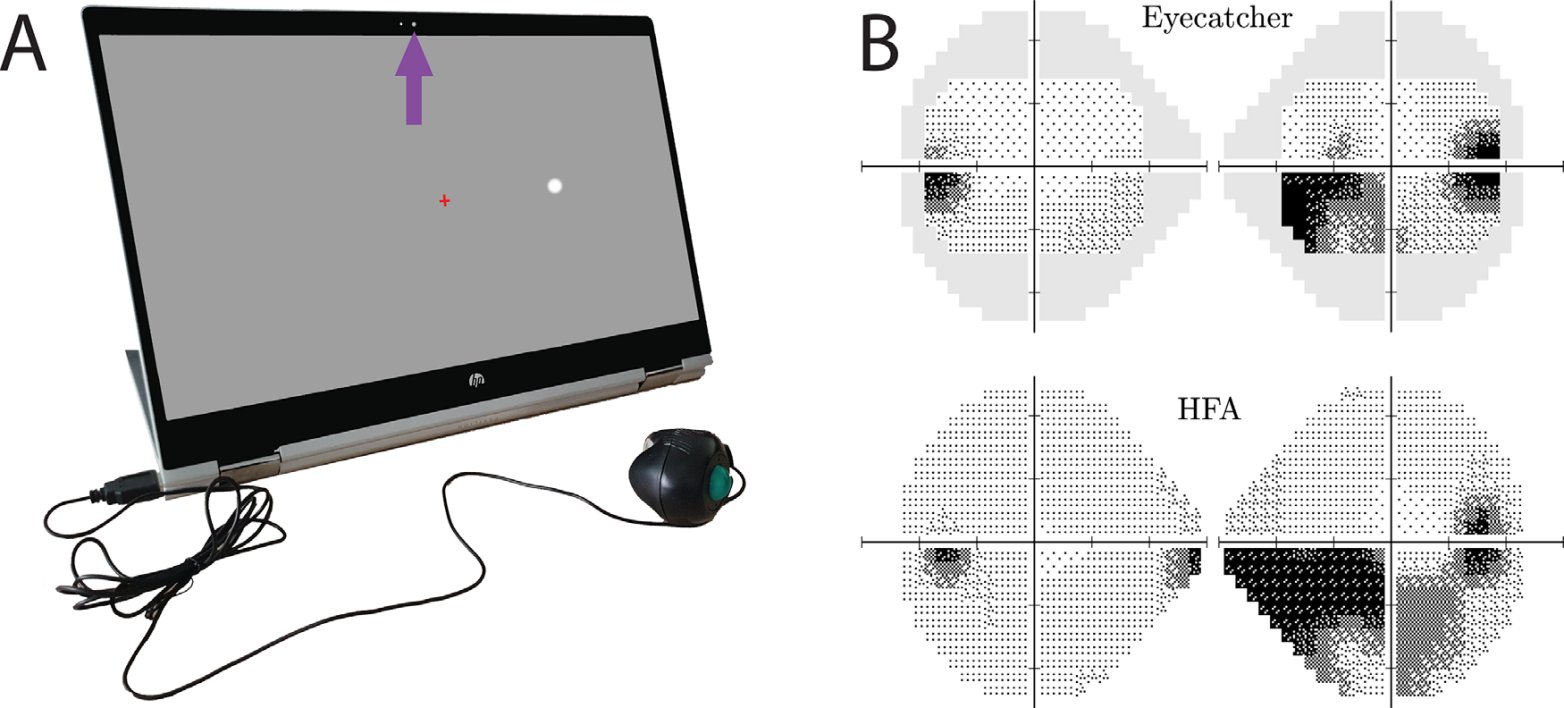
Visual field assessments. (A) Perimetry was performed using the inexpensive screen perimeter shown here (Eyecatcher). Test–retest error was computed by examining differences in mean sensitivity (MS) across repeated Eyecatcher assessments (healthy adults) and between Eyecatcher and a same-day HFA assessment (patients). During the Eyecatcher assessment, live recordings of the participant were made via the screen's front facing camera (*purple arrow*). (B) Example measures of visual field loss from a single participant, with same-patient data from the HFA for comparison. In all cases, only the central 24 points of the 24-2 grid were analyzed when computing MS. Grayscales were generated using MATLAB code available at https://github.com/petejonze/VfPlot.

The seven patients were under ongoing care from an ophthalmologist in the United Kingdom and had an established diagnosis of bilateral primary open-angle glaucoma (*n* = 6) or unilateral secondary glaucoma (*n* = 1). Before participating in the present study, their condition was confirmed by an assessment by a glaucoma-accredited optometrist (P.C.), including a full ocular health check, medical history, logMAR acuity, and standard automated perimetry using the Humphrey Field Analyzer 3 (HFA, Carl Zeiss Meditec, Jena, Germany) with the Swedish Interactive Threshold Algorithm (SITA) Fast 24-2. All patients exhibited best-corrected logMAR acuity < 0.5 in the worse eye, and none had undergone ocular surgery or laser treatment within 6 months prior to participation. Severity of visual field loss,^13^ as measured by HFA mean deviation, varied from –2 dB (early) to –18 dB (advanced), although the majority of eyes exhibited moderate loss (median = –8 dB). An example patient's visual field is shown in Figure 1B.

Healthy adults wore glasses or contact lenses as required, and normal vision was defined as no history of eye disease, binocular best-corrected logMAR acuity ≤ 0.2 (tested with an Early Treatment Diabetic Retinopathy Study chart), binocular best-corrected Pelli–Robson contrast sensitivity ≥ 1.5 logCS (tested with Pelli–Robson chart at 4 m), and a passing score (25 correct of the first 25 plates) on the 38-plate Ishihara pseudoisochromatic test (Handaya, Tokyo, Japan, 2011 edition). Prior experience of perimetry was not recorded, but it is likely that few if any of these participants had undergone a static threshold perimetry assessment previously.

Participants were recruited via advertisements placed in the International Glaucoma Association newsletter (patients) and around City, University of London (healthy adults). The study was approved by the Ethics Committee for the School of Health Sciences, City, University of London (#ETH1819-0532) and was carried out in accordance with the tenets of the Declaration of Helsinki. Written informed consent was obtained from all participants prior to testing.

### Procedure for Visual Field Assessments

All test eyes underwent two monocular visual field assessments within a single session.

#### Normally Sighted Adults

Each of the 42 healthy eyes was assessed using a custom screen perimeter (Fig. 1A), implemented on an HP Pavilion x360 15-inch laptop (HP Inc., Palo Alto, CA). The test was a variant of the Eyecatcher visual field test, which has been described previously,^14^ the source code for which is freely available online (https://github.com/petejonze/Eyecatcher). It was modified in the present work to more closely mimic conventional static threshold perimetry, most notably by employing a Zippy Estimation by Sequential Testing (ZEST) thresholding algorithm,^15^ a central fixation cross, and a button press response. The software was implemented using Psychtoolbox 3,^16^ and we used bit stealing to ensure >10-bit luminance precision,^17^ with extensive photometric calibration to ensure stimulus uniformity across the display (see Kyu Han and Jones^18^ for technical details regarding the calibration method). Unlike conventional perimetry, participants received visual feedback regarding the true stimulus location after each button press. This was a feature that had previously been requested by patients and was intended to keep participants motivated and alert during testing; it was not considered to have affected the findings of the present study substantively. The output of each Eyecatcher assessment was a 4 × 6 grid of differential light sensitivity (DLS) estimates, corresponding to the central 24 locations of a standard 24-2 perimetric grid (Fig. 1B; ±15° horizontal and ±9° vertical). For analysis and reporting purposes, these values were transformed to be on the same decibel scale as the HFA: dB = 10log_10_(3183.1/DLS_cd/m^2^_). A summary measure of visual field sensitivity was computed by mean averaging these 24 DLS_dB_ values, resulting in two mean sensitivity (MS) values per participant (one per test; same eye). Another common summary metric of visual field sensitivity is mean deviation. None of the present findings differed if this metric was used instead of MS (see Supplementary Materials). Test–retest measurement error was quantified as the absolute difference in MS between each test. The two tests were performed consecutively in a single session, with a brief pause of ∼60 seconds between tests.

#### Patients

The 14 patient eyes were assessed only once by Eyecatcher; therefore, to quantify measurement error we also analyzed same-day visual field data from the HFA (SITA Fast 24-2). Note that the results of the two tests were highly correlated (Pearson correlation, *r*_12_ = 0.86; *P* < 0.001) with no significant difference in mean score (repeated measures *t*-test, *t*_13_ = 1.38; *P* = 0.190). For equivalence with Eyecatcher, MS from the HFA assessment was computed by averaging across the central 24 test locations only. Test–retest measurement error was computed as the absolute difference in MS between the two tests (MS_Eyecatcher_ – MS_HFA_).

### Biomarkers of Task Compliance

Seven potential biomarkers of task compliance were considered: gaze variability, head location variability, head rotation variability, mean sadness, mean surprise, blink rate, and mean response latency. These seven were selected based on informal piloting and pragmatism (being easy to implement and computable in real time) and followed an initial assumption that less compliant individuals would be more likely to fidget or exhibit displeasure. These seven metrics were not intended as comprehensive or ideal, however. Other, potentially more informative, biomarkers can be measured with additional hardware (see Discussion section). Also, further variables could have been computed from the present video data, including additional facial expressions (e.g., disgust, contempt, happiness) and more complex head- or eye-movement statistics.^19^

**Figure 2.**
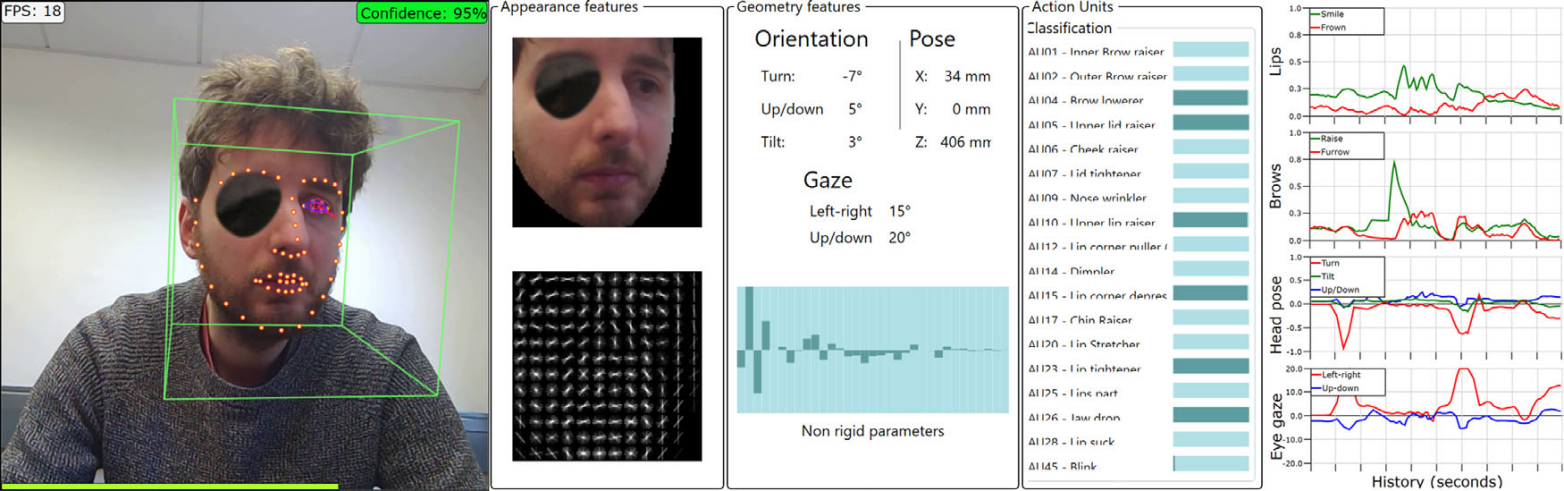
Biomarkers of task compliance. Various biomarkers were computed from raw video footage of the Eyecatcher assessment (recorded using the laptop's built-in webcam). Measures of eye gaze, head pose, and facial expression were extracted using freely available machine learning software (OpenFace 2.0). The data shown here are from author P.R.J. and are for illustration purposes only.

Details of how each variable was computed are given below. In general, however, they were derived primarily from the video footage of a low-budget webcam (the integrated camera of the HP Pavilion x360 15-inch laptop, recorded at 5 Hz with 640 × 480 pixel resolution). As illustrated in Figure 2, data were extracted from the raw video images using OpenFace 2.0, a free machine-learning tool for facial landmark detection, head pose estimation, facial action unit recognition, and eye-gaze estimation.^20^ OpenFace 2.0 uses state-of-the-art techniques, including deep learning, to make fast and accurate decisions, and it has been applied previously to assess dementia,^21^ depression,^22^ and suicidal ideation.^23^ It has also been used to improve automatic speech recognition,^24^ perform video classification,^25^ monitor engagement with e-learning materials,^26^ and inform trauma-recovery regimens.^27^ The raw data for all participants, as extracted by OpenFace 2.0, are available as Supplementary Materials (original video footage not available for reasons of data protection and personal privacy).

#### Gaze Variability

Eye gaze was estimated by OpenFace 2.0 using a constrained local neural fields landmark detector (expected mean absolute error, ∼9 degrees^20^). This yielded one vector of 〈*x, y*〉 gaze coordinates (in degrees visual angle) per video frame; for example, a 2 × 1200 matrix of values was produced in a typical 4-minute test, given the 5-Hz sampling rate at which the camera was recorded. Gaze variability was quantified as the median absolute distance of every gaze point from every other gaze point (i.e., Rousseeuw and Croux's *S_n_* factor^28^). This is a non-parametric and highly robust measure of dispersion, which, unlike bivariate contour ellipse area,^29^ does not require unrealistic assumptions of normality and is not distorted by small numbers of statistical outliers.^30^ The final outcome from each visual field assessment was a single scalar variable, given mathematically by

(1a) $$\mathrm{Gaze}\;\mathrm{variability}=c_{n}\underset{i=1:n}{\mathrm{med}}\left\{\underset{j\neq i}{\mathrm{med}}\left|d_{ij}\right|\right\}$$

where *c_n_* is a bias correction factor for finite sample sizes (which for present purposes can be assumed to equal unity), *n* is the number of video frames, and *d_ij_* is the Euclidean distance between the estimated gaze coordinates in the *i*th and *j*th video frames:

(1b) $$d_{ij}=\sqrt{{\left(x_{i}-x_{j}\right)}^{2}+{\left(y_{i}-y_{j}\right)}^{2}}$$

#### Head Location Variability

The location of the head (head pose translation) was estimated by OpenFace 2.0 using a speed-optimized convolutional experts constrained local model.^20^ This yielded one vector of 〈*x*, *y*, *z*〉 location coordinates (in millimeters) per video frame. Variability in head location was computed in the same manner as gaze variability:

(2) $$\begin{array}{ccc} & & \;\mathrm{Head}\;\mathrm{location}\;\mathrm{variability}\\ & & \;=c_{n}\underset{i=1:n}{\mathrm{med}}\left\{\underset{j\neq i}{\mathrm{med}}\left\{\sqrt{{\left(x_{i}-x_{j}\right)}^{2}+{\left(y_{i}-y_{j}\right)}^{2}+{\left(z_{i}-z_{j}\right)}^{2}}\right\}\right\} \end{array}$$

#### Head Rotation Variability

The rotation of the head (head pose orientation) was estimated as part of the same head pose pipeline as head location (expected mean absolute error, ∼3 degrees).^20^ This yielded one vector of 〈*yaw*, *pitch*, *roll*〉 values (in degrees) per video frame. Variability in head rotation was computed in the same manner as gaze variability and head location variability:

(3) $$\begin{array}{ccc} & & \;\mathrm{Head}\;\mathrm{rotation}\;\mathrm{variability}\\ & & \;=c_{n}\underset{i=1:n}{\mathrm{med}}\left\{\underset{j\neq i}{\mathrm{med}}\left\{\sqrt{{\left(Y_{i}-Y_{j}\right)}^{2}+{\left(P_{i}-P_{j}\right)}^{2}+{\left(R_{i}-R_{j}\right)}^{2}}\right\}\right\} \end{array}$$

#### Mean Sadness

OpenFace 2.0 recognizes facial expressions by using linear kernel Support Vector Machines to detect the intensity and presence of 18 discrete facial action units (AUs), each corresponding to entries in the classic Facial Action Coding System (FACS) taxonomy of human facial movements.^31^ The primary output was 18 positive scalar intensity values, where 0 indicates the complete absence of a given AU. Following established convention,^32^ sadness was estimated by summing AU4 (brow lowerer) and AU15 (lip corner depressor). This yielded one value (in arbitrary units of intensity) per video frame. Mean sadness was computed simply as the arithmetic mean of these values:

(4) $$\mathrm{Mean}\;\mathrm{sadness}=\frac{1}{n}\sum_{i=1}^{n}\left[AU4_{i}+AU15_{i}\right]$$

#### Mean Surprise

Mean surprise was estimated in the same manner as mean sadness. Following established convention,^32^ surprise was estimated by summing AU1 (inner brow raiser), AU2 (outer brow raiser), AU25 (lips part), and AU26 (jaw drop):

(5) $$\begin{array}{ccc} \mathrm{Mean}\;\mathrm{surprise} & = & \frac{1}{n}\sum_{i=1}^{n}[AU1_{i}+AU2_{i}\\ & & +AU25_{i}+AU26_{i}] \end{array}$$

#### Blink Rate

The presence of a blink is encoded by AU45 of the FACS taxonomy. Blink rate was therefore estimated in the same manner as sadness and surprise:

(6) $$\mathrm{Blink}\;\mathrm{rate}=\frac{1}{n}\sum_{i=1}^{n}AU45_{i}$$

#### Mean Response Latency

Unlike the other six biomarkers, response latency was not derived from the video footage. It was instead computed simply as the difference (in seconds) between the onset of a given stimulus presentation and the participant's button press response (τ). This was recorded only for trials where the participant responded to the stimulus. Mean response latency was computed simply as the arithmetic mean of these values:

(7) $$\begin{array}{ccc} \mathrm{Response}\;\mathrm{latency} & = & \frac{1}{\sum_{i=1}^{N}\left[\tau_{i}\neq NULL\right]}\\ & & \times \sum_{i=1}^{N}\tau_{i}\left[\tau_{i}\neq NULL\right] \end{array}$$

where *N* is the total number of trials (i.e., stimulus presentations).

#### Composite of All Biomarkers

A composite of all seven individual biomarkers was computed by standardizing each measure as a *z*-score, and then taking their weighted linear sum:

(8a) $$\mathrm{Composite}=\sum_{i=1}^{7}\omega_{i}Z\left(X_{i}\right)$$

where the weights, ω*_i_*, were proportional to the correlation coefficient (ρ) between each measure and observed performance (see Fig. 2 for values), normalized so that they summed to one:

(8b) $$\omega_{i}=\frac{\rho_{i}}{\sum_{j=1}^{7}\rho_{j}}$$

Note that the decision to average all seven values in this way was an intentionally crude approach to avoid overfitting the available data. With a larger dataset, however, it would be possible to use a machine learning approach to determine the optimal combination of parameters required to detect lapses in concentration (see Discussion section).

### Methods of Analysis

#### Association with Overall (Test–Retest) Measurement Error

The association between each biomarker and overall (test–retest) measurement error was assessed using ordinary linear correlation (Pearson product–moment correlation). Measurement error was quantified as the absolute difference in MS between two monocular visual field assessments: either two Eyecatcher assessments (healthy eyes) or one Eyecatcher assessment and one HFA assessment (patient eyes). This resulted in one scalar estimate of measurement error per eye, in units of dB. Independently, seven potential biomarkers were computed as detailed previously, each yielding one scalar value per assessment (Eyecatcher assessments only). For healthy eyes (which were assessed by Eyecatcher twice), each pair of biomarker estimates was mean-averaged to produce a single value. For patient eyes (which were assessed by Eyecatcher once and the HFA once), only the biomarker estimates from the Eyecatcher assessment were available. Any conspicuous non-compliance on the HFA test would therefore not have been detected, and so the predictive power of the various biomarkers may have been underestimated in patients.

#### Association with Trial-by-Trial Measurement Error

To examine whether each biomarker could also be used in real time to identify lapses in concentration during testing, the following trial-by-trial analysis was performed.

For each visual field assessment (Eyecatcher only; all eyes, healthy and glaucoma together), we took the final set of 24 pointwise (DLS) estimates as the best guess of each participant's true threshold at each location. We then extracted all individual trials where the stimulus intensity was more than 3 dB below threshold (brighter) at a given location (i.e., all visible trials). The −3 dB criterion represents a doubling of stimulus intensity (in cd/m^2^) and was intended to be well above the slope of a typical frequency-of-seeing curve, which is on the order of ∼1 dB for healthy visual field locations.^33^^,^^34^ Note, however, that slopes can vary among individuals and can increase to 10 dB or more for very severely affected locations.^33^^,^^34^ This, together with the fact that the threshold estimates themselves are subject to non-trivial measurement error,^35^^,^^36^ means that the –3 dB threshold should only be taken as indicative, and we cannot guarantee that every stimulus presented below this cutoff was always visible. We do not anticipate, however, that the present findings would differ substantively if a somewhat different criterion had been used. To the extent that “invisible” stimuli were inadvertently included in analyses, any of the statistical associations that we report were likely underestimated.

This procedure yielded 4598 trials (of 13,867 total). Of these, we termed a failure to respond as a miss (false negative), and a successful response was termed a hit (true positive). Ideally, the hit rate, *P*(hit), for such suprathreshold stimuli should equal 1, whereas missed stimuli (4.6%) we took as being indicative of a lapse in concentration. Finally, we examined how well each biomarker predicted hits and misses on each trial. To do this, we recomputed each biomarker for each of the 4598 stimulus presentations, using only video data from that trial, and from the 20 frames (4 seconds) directly preceding it. Note that the choice of 20 preceding frames was arbitrary; other values were never attempted, but we have no reason to expect that other similar values would not yield qualitatively similar results. Note also that the response latency biomarker was not analyzed, as this was by definition “null” for missed trials and so contained no useful information regarding missed stimuli.

## Results

### Predicting Overall (Test-by-Test) Measurement Error

**Figure 3.**
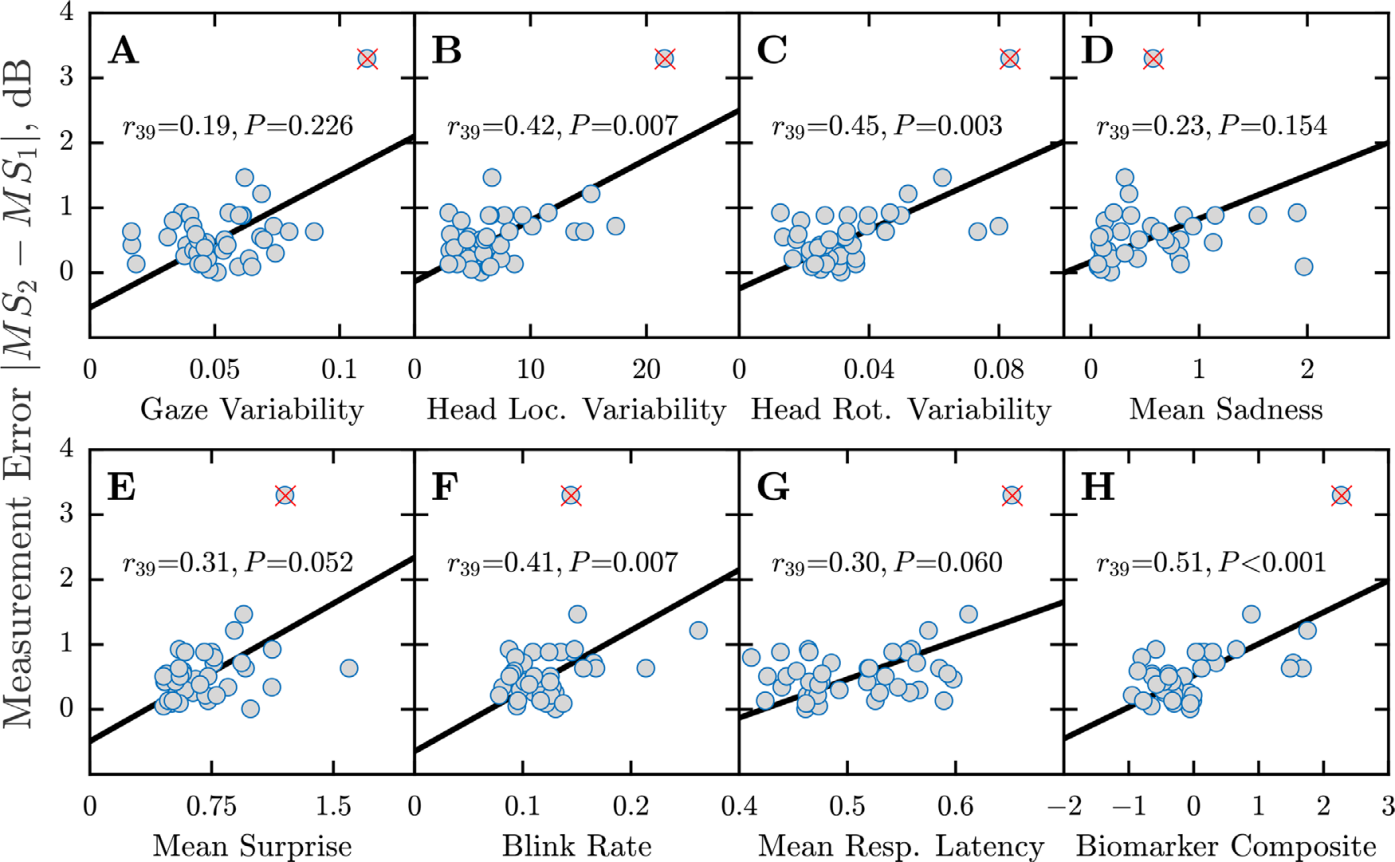
Overall test-retest data from healthy eyes. Each panel shows visual field measurement error (absolute test–retest difference in mean sensitivity) as a function of seven potential biomarkers of task compliance (A–G), as well as a function of a composite measure computed as the linear-weighted sum of all seven individual biomarkers (H). See Methods section for technical details on how each variable was computed. Markers show the raw measurements for individual eyes. The marker with a *red cross* was excluded from all analyses as a possible statistical outlier. However, all *P* values were smaller if this point was included. Black lines show geometric mean regression slopes. Figures within each panel show correlation statistics.

Results for healthy eyes are shown in Figures 3A to 3G, broken down by biomarker. Considered in isolation, three of the seven biomarkers (head location variability, head rotation variability, and blink rate) were significantly associated with overall (test–retest) measurement error (*P* = 0.003–0.007). By inspection, the four other biomarkers exhibited possible trends, with at least two associations close to reaching statistical significance (*P* = 0.052–0.060). Put simply, the tests with lowest test–retest variability tended to be those during which individuals moved their eyes least (*P* = 0.226), moved their head least (*P* = 0.007, *P* = 0.003), exhibited least sadness (*P* = 0.154) or surprise (*P* = 0.052), blinked least (*P* = 0.007), and responded consistently quickly (*P* = 0.060). Furthermore, when all seven individual biomarkers were averaged together, a single composite variable was even more highly associated with measurement error than any single biomarker alone (Fig. 3H; *P* < 0.001, *r* = 0.51).

**Figure 4.**
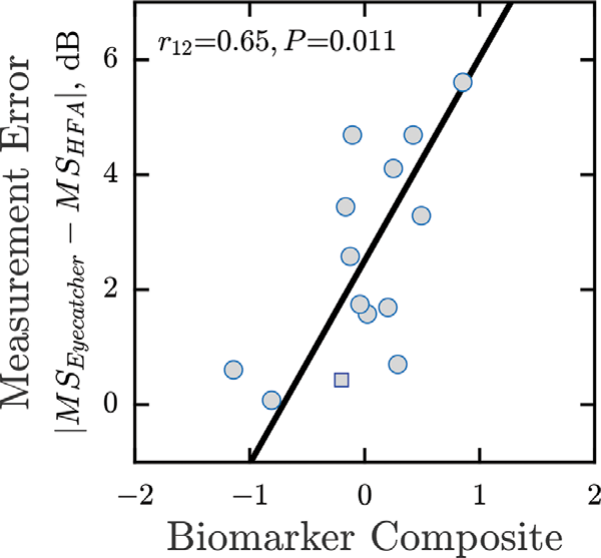
Overall test-retest data from 14 eyes from glaucoma patients; same format as Figure 3H. Note that in this instance, MS_1_ was measured using the HFA (not the screen perimeter). However, in practice the values from the two tests were robustly correlated (Pearson correlation, *r*_12_ = 0.86; *P* < 0.001), and any deviation between the two would likely only serve to minimize (add noise to) any of the effects reported in the present work. The *square marker* indicates the fellow eye from the one patient with unilateral secondary glaucoma for which the visual field was within normal limits.

We used analogous data for the 14 eyes from glaucoma patients to confirm the repeatability of these results and to ensure that they generalize to patients (Fig. 4). As with normally sighted individuals (shown previously in Fig. 3H), there was a statistically significant positive association between the composite biomarker metric and test–retest variability (*P* = 0.011). The range of measurement errors observed was much greater in patients, however (Fig. 4; note the difference in *y*-axis scale), consistent with previous reports of higher measurement variability in eyes with visual field loss.^36^ Possibly owing to the small sample size, none of seven individual biomarkers alone reached significance in patients (*P* > 0.05; data not shown). None of the biomarkers was correlated with false-positive rates (mean *P* = 0.206) or false-negative rates (mean *P* = 0.565) on the HFA.

### Predicting Trial-by-Trial Lapses

The foregoing analyses suggest that the proposed biomarkers are associated with the overall reliability of a visual field assessment. Such measures could potentially complement or replace existing reliability metrics, such as fixation stability, false-positive rates, or false-negative rates,^37^^,^^38^ the latter having been shown to “depend more on visual field status than on the patient attentiveness.”^39^ Even better, however, would be if these biomarkers could also be used in a more granular fashion to detect lapses in concentration in real time, during the test. To examine whether this is possible, we performed the trial-by-trial analysis detailed in the Methods section.

**Figure 5.**
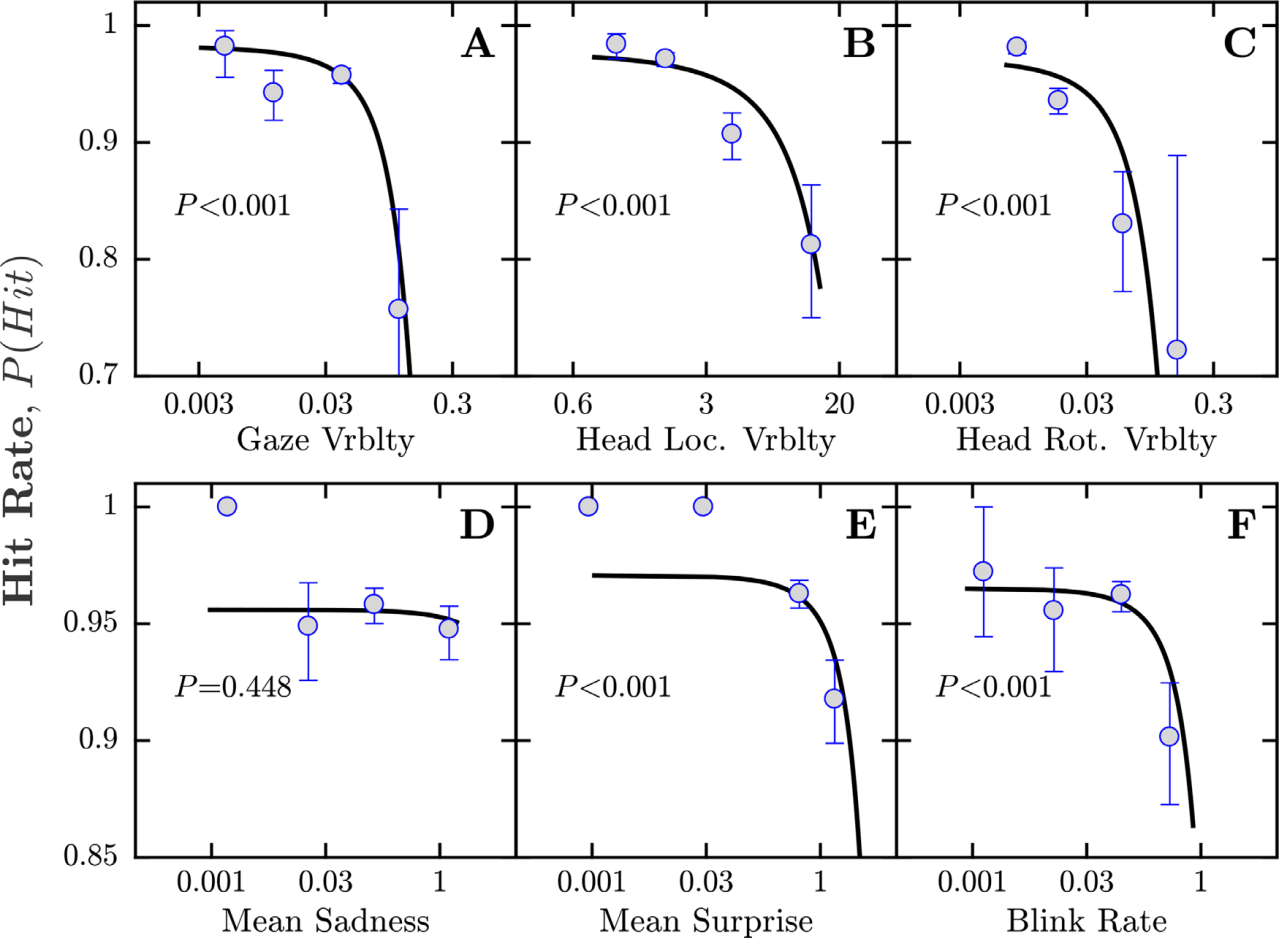
Results of trial-by-trial analyses examining the proportion of easily visible (−3 dB or brighter) stimuli that were correctly responded to as a function of biomarker magnitude (biomarkers computed using only data from the preceding 4 seconds of each given trial; see main text for details). We considered a failure to respond to such stimuli as an obvious lapse in concentration. Markers represent mean hit rate [± 95% confidence intervals] for binned data, aggregated across all participants (binning performed by MATLAB's “histcounts” function, separating biomarker values into four log-spaced bins). Black lines and *P* values represent the result of logistic regressions fitted to the raw binary (hit/miss) data (not to the displayed markers). Note that these curves are plotted on a log *x*-axis, although tickmark values are shown in the original linear units, and all analyses were performed on the original, untransformed data. *P* values give the results of χ² tests, examining whether the logistic model fits the data significantly better than a constant model.

The results are shown in Figure 5. They indicate that at least five of the six biomarkers were predictive of trial-by-trial lapses, with the hit rate, *P*(hit), for visible (>3 dB suprathreshold) stimuli decreasing progressively as a function of biomarker magnitude (logistic regression, *P* < 0.001). The only exception to this was mean sadness (*P* = 0.448), although by inspection this too exhibited a possible weak association (Fig. 5D). In short, the results in Figure 5 indicate that local biomarkers, computed using video frames from a single trial (and the 20 frames immediately preceding stimulus onset), were predictive of whether or not a participant made a “lapse” (false negative response) on that trial.

## Discussion

Data from both normally sighted young adults and older glaucoma patients indicated that autonomous biomarkers of task compliance are associated with measurement error during visual field assessments. The association was greatest when multiple biomarkers were considered in combination and was true in terms of the overall reliability of a test (with multiple biomarkers associated with test–retest repeatability), as well as with individual trials (with multiple biomarkers associated with failures to respond to visible targets). What is particularly remarkable is that the biomarkers that we considered here can be computed in real time, using only an ordinary webcam and without the need for a powerful computer (e.g., the hardware inside of an ordinary smartphone or tablet computer is sufficient).

It should be noted that many of the associations observed, although statistically significant, would not generally be regarded as strong.^40^ For example, the composite of all seven biomarkers (Figs. 3H, 4) explained only ∼25% of the variation in overall test–retest reliability. However, even this finding we take as encouraging, given that this is only a preliminary investigation of feasibility and given that this essentially represents “free” information: measurements that can be made in the background without requiring existing perimetric protocols to be altered in any way and without extending assessment durations, or the demands placed on patients. This is in contrast to traditional compliance metrics, such as false-negative rate estimation, which often require additional catch trials,^39^ thus prolonging the test and potentially increasing the risk of fatigue or lapses in concentration.

We were particularly encouraged by the association between real-time, trial-by-trial estimates of each biomarker and performance (lapses) on specific trials (Fig. 5). Thus, although a measure of overall reliability can be helpful for flagging poor-quality assessments post hoc, being able to monitor task compliance in real time could be even more useful as a way of proactively reducing measurement error, “at source”. One straightforward way to do this might be to use anomalous biomarker estimates to trigger automated feedback, encouraging the patient to keep going and remain vigilant. Another complementary option would be to factor the estimated reliability of each data point (i.e., each button-press response, or absence thereof) into the underlying psychophysical algorithm. To see how this might be achieved, note that most modern perimeters already use probabilistic (maximum likelihood) algorithms to estimate sensitivity.^15^ These work, fundamentally, by computing the the likelihood of each possible sensitivity value (i.e., each possible psychometric function), given the observed sequence of responses. This in turn is proportional to the likelihood of having observed a particular pattern of responses, given each possible sensitivity value:

(9a) $$p\left(r|\left\{x,\psi \right\}\right)=\prod_{i=1}^{n}p\left(r_{i}|\left\{x_{i},\psi \right\}\right)$$

where $x_{i}$ is the stimulus level on trial $i,r_{i}$ is the participant's response, *n* is the total number of trials, and ψ is the set of all possible psychometric functions. As we have described previously elsewhere,^41^ trial-by-trial information regarding compliance can be integrated into Equation 9a simply by modifying the likelihood function, such that the participant's response on each trial is weighted by the estimated reliability of that response:

(9b) $$p^{\alpha }\left(r|\left\{x,\psi \right\}\right)=\prod_{i=1}^{n}\left[p{\left(r_{i}|\left\{x_{i},\psi \right\}\right)}^{\alpha \left(\theta_{i}\right)}\right]$$

were α(θ*_i_*) is the estimated compliance on trial *i*, transformed to be a value between 0 and 1. When α(θ*_i_*) = 0 (estimated complete non-compliance), that trial is given zero weight—the response is effectively ignored and the likelihood function remains unchanged. When α(θ*_i_*) = 1 (estimated perfect compliance), the trial information is integrated into the likelihood function exactly as per usual. At intermediate values of α(θ*_i_*), trials are given partial credit. This weighting approach has been suggested in other domains as a way of adjusting for anomalous statistical data^42^ and has been shown to provide a consistent and efficient likelihood estimate while preserving the same first-order asymptotic properties of a genuine likelihood function. Our expectation is that such a probabilistic weighting approach would yield more reliable likelihood estimates than current methods, which naïvely assume that every response from every participant is equally informative.

Under this proposed scheme, all other aspects of the psychophysical algorithm remain unchanged. It would therefore still be possible, for example, to compute expected entropy, which can be used both to determine the most informative stimulus to present on the next trial^43^ and to ascertain when a given level of measurement certainty has been attained.^44^ This would mean that more compliant participants would be required to complete fewer trials, whereas non-compliant participants may be asked to complete additional trials in order to reach a given level of data quality. (Note that this would prolong some tests but, unlike with the use of catch trials discussed previously, would not prolong all tests indiscriminately, and any additional trials would contribute directly toward improving measurement precision.) A conspicuously non-compliant participant might never reach the stopping criterion within a prescribed number of trials and so would be scored as “did not complete.” However, this seems preferable to the present situation, where such individuals produce spurious data that must be excluded post hoc by clinicians, often using unstandardized criteria.

### Previous Literature

The present study is not the first to consider ways of monitoring task compliance in perimetry. For example, Henson and Emuh^45^ used near-infrared eye tracking and found that certain eye-tracking parameters (pupil miosis and fatigue wave amplitude) were related to vigilance (probability of seeing a stimulus) in glaucoma patients. Similarly, Wang et al*.*^46^ examined blinks and found that the probability of seeing was reduced when blinks overlapped with a stimulus presentation, although there was no association between overall blink rates and threshold variability. This latter finding is *prima facie* inconsistent with the present work. However, even in the present work, the association between blink rate and threshold variability was significant only in healthy eyes (not glaucoma), and in general no single biomarker was strongly associated with overall test-retest measurement error—with a more robust association being observed when multiple biomarkers were combined together. Outside of ophthalmology, the present study is also not the first to examine eye movements,^47^ head movements,^48^ or facial expressions^49^^,^^50^ as ways of determining whether an individual is alert and engaged.

What is novel about the present work is that we consider a wide range of different metrics, all of which can be derived autonomously, in real time and without the need for expensive or complex hardware. We also introduce a way of integrating such measurements into existing perimetric routines and demonstrate that key findings can be replicated across independent datasets.

With respect to existing measures of reliability in perimetry, a number of techniques have been explored previously.^37^^,^^38^ These include (1) the Heijl–Krakau method of detecting fixation loss by measuring (false-positive) responses to stimuli presented to the blind spot; (2) measuring false positives as responses made after the end of the current response window (and/or just after the onset of the next stimulus); and (3) measuring false negatives as failures to respond to stimuli more intense than those responded to previously. These existing measures are far from complete in capturing non-compliance and often “depend more on visual field status than on the patient attentiveness.”^39^ It is tempting to speculate whether the sorts of biomarkers described in the present study could potentially complement or replace these existing reliability metrics. For example, Ishiyama and colleagues^51^ advocated the use of gaze tracking metrics, similar to the gaze variability biomarker used here, and compared its utility to the traditional metrics described above. To some extent, however, we regard such comparisons as moot. These conventional metrics are concerned purely with the post hoc identification of bad data, and our goal is to prevent poor quality assessments from occurring in the first place. To achieve this requires us to be able to continuously monitor compliance throughout the test and to do so in a way that is fast, automatic and can be linked directly to the underlying psychophysical algorithm. In this sense, the present work is perhaps most closely related not to previous explorations of perimetric measures of reliability but to acuity card testing in infants, where pediatric clinicians routinely use a range of facial and body expressions to gauge the child's interest^11^ and then use this information to dynamically modify the assessment protocol accordingly (e.g., ignoring suspicious responses, pausing the test momentarily). The present work is encouraging in that it suggests that it may be possible to exploit modern digital technologies to provide a similar level of care and attention to the assessments of adult glaucoma patients.

More generally, the present work can also be viewed in the context of attempts more widely to replace or augment human technicians (e.g., to facilitate home monitoring^12^ or free up manpower in clinics^9^). Some of the techniques used in the present study have also been used elsewhere to make visual field testing more comfortable and physically accessible to patients (e.g., by using head and eye tracking to obviate the need for chin rests and fixation targets^14^). It is also interesting to note that some of the biomarkers considered here (e.g., blinking, unsteady fixation) have also been shown to provide direct indices of the presence and magnitude of various ophthalmic pathologies.^52^

### Study Limitations

The present work was intended to demonstrate feasibility only. The methods described are not intended as optimal or comprehensive.

In terms of the specific biomarkers employed, other measures have been proposed as possible indicators of whether an individual is alert and engaged.^53^ These include postural instability,^41^ movements of the upper body and torso,^54^ skin conductance/temperature,^55^ heart rate,^56^ vocal expressions,^57^^,^^58^ electroencephalogram-based neural activity,^59^^–^^61^ functional magnetic resonance imaging blood-oxygen-level-dependent responses,^62^^–^^65^ and pupil dilation.^56^^,^^66^^,^^67^ These additional biomarkers are not mutually exclusive, and in future it would be instructive to examine whether greater sensitivity could be achieved by incorporating such additional measures into the present battery. Unlike those used in the present work, however, many of these additional biomarkers require specialized hardware and may be less practical for everyday clinical applications.

In terms of precisely how each biomarker was computed and how information from multiple biomarkers was combined, care was taken not to overfit the present data. For example, it is extremely likely that better (or worse) performance could have been obtained by using alternative techniques to summarize the data (e.g., alternative measures of dispersion or central tendency), by tweaking key parameters (e.g., number of frames analyzed in the trial-by-trial analysis), or by attempting to determine the most predictive combination of parameters (e.g., through the application of machine learning). Ultimately, however, a far larger dataset would be required in order to solve such questions of optimization, to which end all of the data from the present study have been made available as Supplementary Materials.

Equally, it is likely that even more sensitive biomarkers could be obtained in the future through improved hardware or more sophisticated computer vision algorithms. It is noteworthy, for example, that the biomarkers in the present study were derived from an integrated laptop camera, sampled at only 5 Hz and 640 × 480 pixel resolution. Greater spatiotemporal fidelity may allow us to capture more subtle changes in head pose or facial expression or to track rapid eye movements (saccades). With accurate enough gaze tracking, it might even be possible to explore whether associations between biomarkers and measurement reliability differ as a function of stimulus location. Against this, however, must be balanced practical considerations, such as the computational power required to process and store high-resolution video data in near real time. Thus, automated measures of compliance would be particularly beneficial in situations such as home monitoring where a technician cannot be present to observe the patient. Such benefits would be diminished, however, if complex or expensive equipment was required in order to carry out the assessment in the first place.

In terms of study design, it will be necessary in the future to assess the present techniques in a larger and more representative cross-section of patients. Thus, the present study examined only young university students and a self-selecting cohort of patients. The patients in particular appeared highly motivated and were likely more compliant than the typical individual seen in a busy glaucoma clinic (e.g., HFA median [IQR] false-positive and false-negative rates were 1% [0–3%] and 4% [0–8%], respectively). It may even be that stronger associations are observed in more heterogeneous populations (i.e., exhibiting a wider spread of measurement error^68^), and it would be instructive to examine how robustly the present biomarkers are able to identify the most compromised visual field assessments. Likewise, it is well established that test variability increases with eccentricity,^69^^–^^72^ whereas testing in the present study was limited to the central 15°. Future studies might use a larger screen to consider how well biomarkers correlate with a greater dynamic range of measurement variability at more peripheral test locations.

In future, it will also be important to examine patients with a wide range of severities. It is known that the depth of glaucomatous defect can be a substantive confound in perimetry, both for existing reliability metrics^37^ and estimates of sensitivity.^36^ It is likewise conceivable that a patient with advanced loss may appear more restless or despondent, potentially affecting several of the proposed biomarkers independent of task performance. The precise relationship between observed biomarkers and task compliance will therefore need to be quantified both rigorously and sensitively.

## Conclusions

Using only an ordinary webcam, it is possible to derive real-time measures of task compliance during visual field assessment, and these can be used to identify unreliable assessments and/or unreliable responses within an assessment. In the long term, such autonomous measures could facilitate the creation of more intelligent and accessible forms of vision assessment: assessments in which “compliant” individuals can be processed even more rapidly than at present, but wherein individuals who might otherwise struggle to complete an automated test will be given the additional time, care, and attention required to ensure robust, clinically useful data.

## Supplementary Material

Supplement 1
