# Supplementary material for: The Human Touch: Using a Webcam to Autonomously Monitor Compliance During Visual Field Assessments
Source: Transl Vis Sci Technol. 2020 Jul 20;9(8):31. doi: 10.1167/tvst.9.8.31 (PMC7422775; doi:10.1167/tvst.9.8.31)
Supplement: Supplement 1 [file tvst-9-8-31_s001.pdf]

Supplemental Material: The human touch: Using a webcam to autonomously monitor compliance during visual field assessments

Pete R. Jones, Giorgia Demaria, Iris Tigchelaar, Daniel S. Asfaw, David F. Edgar, Peter Campbell, Tamsin Callaghan, David P. Crabb

1. Using MD instead of MS as the primary outcome measure

In the present study, the primary summary metric of VF sensitivity was Mean Sensitivity (MS). This was computed by mean averaging all of the 24 DLS$_{dB}$ values measured by Eyecatcher or the HFA.

Another common summary metric in perimetry is Mean Deviation (MD), which expresses the average divergence from age-corrected population norms, with more negative values indicating greater overall VF loss. Clinically, MD is often used for monitoring visual field decline due to glaucoma. This more complex metric was felt unnecessary for the present work, as we were primarily concerned with examining within-subject (test-retest) differences, and also since most of our observers were normally sighted.

For completeness, however, we also re-ran all of the key analyses using MD as the primary outcome measure. MD was computed using a previously reported age-corrected normative values [*Heijl, Lindgren, and Olsson (1987), Archives of Ophthalmology, 105(11):1544-1549*], and with both Eyecatcher and the HFA MD was computed using only the 24 central grid locations (i.e., those points tested by both).

Using MD rather than MS resulted in no qualitative changes in study findings. As shown in **Fig S1**, the overall biomarker composite was still significantly associated with overall measurement error ($r_{44} = 0.44$, $P = 0.004$; versus $r_{44} = 0.51$, $P < 0.001$ in main manuscript), though for some individual biomarkers the association appeared somewhat greater (e.g., Gaze Variability) or weaker (e.g., Head Location Variability, Head Rotation Variability, Mean Sadness). In glaucoma patients, the correlation between Eyecatcher and HFA remained largely unchanged (*Pearson Correlation*; MD: $r_{12} = 0.87$, $P < 0.001$; MS: $r_{12} = 0.86$, $P < 0.001$).

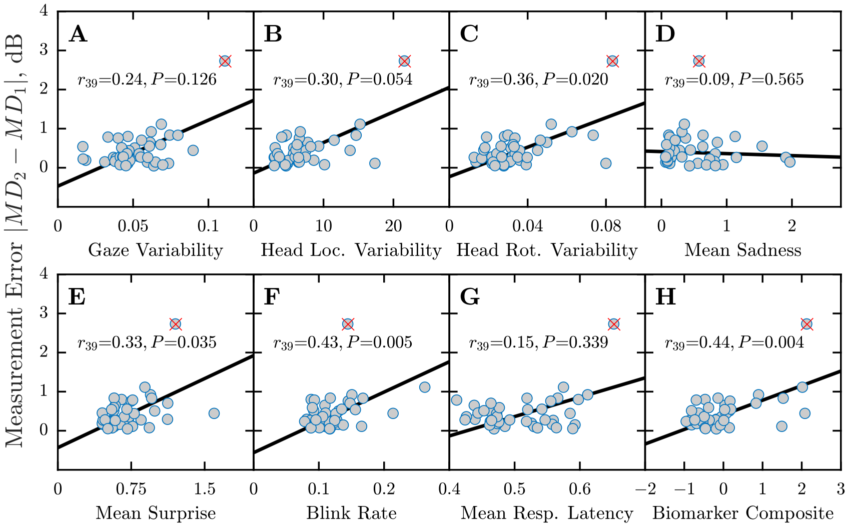

Fig S1. Recomputed version of Figure 3 (main manuscript), using Mean Deviation (MD) rather than Mean Sensitivity (MS) as the outcome measure.
